# Mobile Phone Apps for Quality of Life and Well-Being Assessment in Breast and Prostate Cancer Patients: Systematic Review

**DOI:** 10.2196/mhealth.8741

**Published:** 2017-12-04

**Authors:** Esther Rincon, Francisco Monteiro-Guerra, Octavio Rivera-Romero, Enrique Dorronzoro-Zubiete, Carlos Luis Sanchez-Bocanegra, Elia Gabarron

**Affiliations:** ^1^ Department of Psychology and Pedagogy Universidad San Pablo Centro de Estudios Universitarios Alcorcón (Madrid) Spain; ^2^ Salumedia Tecnologias Seville Spain; ^3^ Department of Electronic Technology Universidad de Sevilla Seville Spain; ^4^ Department of Architecture and Computer Technology Universidad de Sevilla Seville Spain; ^5^ Norwegian Centre for eHealth Research University Hospital of North Norway Tromsø Norway

**Keywords:** cancer, mHealth, app, mobile phone, quality of life, well-being

## Abstract

**Background:**

Mobile phone health apps are increasingly gaining attention in oncological care as potential tools for supporting cancer patients. Although the number of publications and health apps focusing on cancer is increasing, there are still few specifically designed for the most prevalent cancers diagnosed: breast and prostate cancers. There is a need to review the effect of these apps on breast and prostate cancer patients’ quality of life (QoL) and well-being.

**Objective:**

The purposes of this study were to review the scientific literature on mobile phone apps targeting breast or prostate cancer patients and involving QoL and well-being (anxiety and depression symptoms) and analyze the clinical and technological characteristics, strengths, and weaknesses of these apps, as well as patients’ user experience with them.

**Methods:**

We conducted a systematic review of peer-reviewed literature from The Cochrane Library, Excerpta Medica Database, PsycINFO, PubMed, Scopus, and MEDLINE to identify studies involving apps focused on breast and/or prostate cancer patients and QoL and/or well-being published between January 1, 2000, and July 12, 2017. Only trial studies which met the inclusion criteria were selected. The systematic review was completed with a critical analysis of the apps previously identified in the health literature research that were available from the official app stores.

**Results:**

The systematic review of the literature yielded 3862 articles. After removal of duplicates, 3229 remained and were evaluated on the basis of title and abstract. Of these, 3211 were discarded as not meeting the inclusion criteria, and 18 records were selected for full text screening. Finally, 5 citations were included in this review, with a total of 644 patients, mean age 52.16 years. Four studies targeted breast cancer patients and 1 focused on prostate cancer patients. Four studies referred to apps that assessed QoL. Only 1 among the 5 analyzed apps was available from the official app store. In 3 studies, an app-related intervention was carried out, and 2 of them reported an improvement on QoL. The lengths of the app-related interventions varied from 4 to 12 weeks. Because 2 of the studies only tracked use of the app, no effect on QoL or well-being was found.

**Conclusions:**

Despite the existence of hundreds of studies involving cancer-focused mobile phone apps, there is a lack of rigorous trials regarding the QoL and/or well-being assessment in breast and/or prostate cancer patients. A strong and collective effort should be made by all health care providers to determine those cancer-focused apps that effectively represent useful, accurate, and reliable tools for cancer patients’ disease management.

**Trial Registration:**

PROSPERO CRD42017073069; https://www.crd.york.ac.uk/PROSPERO/display_record.asp?ID= CRD42017073069 (Archived by WebCite at http://www.webcitation.org/6v38Clb9T)

## Introduction

The number of new cancer cases diagnosed every year worldwide is rapidly rising from 14.1 million in 2012 to well over 20 million predicted by 2030 [1]. Of those, breast and prostate cancers are the most prevalent diagnosed in women and men, respectively [1]. It should be noted that around 30% to 40% of these cancer patients suffer from psychological distress (anxiety and depression symptoms commonly reported) as has been mentioned previously by a meta-analysis comprising 94 studies and 14,078 cancer patients [2]. This emotional distress has been associated with poorer quality of life (QoL) [3]. Well-being, QoL, and treatment satisfaction in breast and prostate cancer patients could be monitored by ubiquitous technologies such as mobile phone health apps, which can provide useful data to reflect on therapy work [4] and thereby improve patients’ well-being.

Mobile phone health apps have the potential to revolutionize psychological science because they can collect behavioral data [5] and behavioral information with great ecological validity [6], facilitating high-frequency assessments and more objective data collection [7]. These apps can also potentially empower patients, promoting behavior changes, facilitating self-monitoring of symptoms [8], improving their educational level [9], and allowing patients the feeling of being in contact with their health care team [10].

Apps are widely used by professionals and patients, and attention to them in health care environments is increasing daily [9]. However, there are some important concerns about their use. Because of the large number of health care apps available, patients could get overwhelmed, encountering difficulties in finding the right app or features [11]. Poorly validated information, often created by nonexperts [12], and a lack of updated data [13] have also been mentioned as concerning issues related to health apps. Limited evidence involving these apps in studies [14] and little or no quality control or regulations to guarantee the apps as user-friendly, accurate, or efficacious tools [15] have also been reported.

In several systematic reviews on mobile phone health apps, authors urge different strategies that will result in higher quality evidence for app effectiveness and contents [8,13,16-20]. This would allow us to distinguish apps that subscribe to evidence-based protocols from those that do not [21]. The health care team should have a leading role not only in the review and verification of app contents but also in determining the most reliable ones and in selecting the patients best suited to using them [13]. Therefore, health care providers and organizations should standardize the identification, evaluation, and selection of these mobile health (mHealth) apps to maximize their utility and safety [15].

Attending to patients’ point of view about using mHealth apps, authors have commonly used survey studies to determine the user experience. In general, cancer patients positively value the use of Internet-based technologies for health care management and feel comfortable using them [22]. Breast cancer patients usually use this technology to seek general information, search for therapies or scientific data, and exchange information with other patients [23]. Authors have also pointed out the importance of including customizable functionalities in mobile phone apps in order to manage care-related information so that these features can be easily modified depending on changes in the user’s needs [24]. Other people affected by cancer, such as prostate cancer patients, have shown interest in using apps, indicating apps should be easy to use, tailored to the individual, and include social support [25]. A recent survey of 375 cancer patients reported that about half of the patients (182/375, 48.5%) were willing to send data via an app supporting their oncological treatment and follow-up [4]. Moreover, around two-thirds (125/182, 68.7%) agreed to use these regularly sent data as an ideal complement to the standard follow-up. The most mentioned characteristics that should be included in a cancer-focused app were pseudonymizing, data protection, and feedback from a physician based on the patients’ input [4].

Although mHealth apps could be useful tools for cancer patients [26], there are only a few apps focused on oncological care that support patients during treatment and aftercare [4]. The purposes of this study were to (1) identify evidence-based mobile phone health apps focused on QoL and well-being (anxiety and depression symptoms) in breast and/or prostate cancer patients, (2) recognize their clinical and technological characteristics, (3) categorize their clinical and technological strengths and weaknesses, and (4) determine patients’ user experience (satisfaction level and comments regarding the apps used).

## Methods

### Overview

We developed a systematic search strategy to detect all relevant studies involving the use of mobile phone apps for QoL and/or well-being (anxiety and depression symptoms) in breast and/or prostate cancer on July 12, 2017. Once we determined these studies, we searched the identified apps on the online market to describe them. The systematic research protocol is registered at PROSPERO [CRD42017073069].

### Reviewing the Scientific Literature

#### Selection Criteria

Articles were considered potentially relevant if they were trials or peer-reviewed studies published between January 1, 2000, and July 12, 2017, including a smartphone app focused on QoL and/or well-being (anxiety and depression symptoms) used by breast and/or prostate cancer patients.

We considered a smartphone “a mobile phone with Internet connectivity and the ability to download and run third-party software apps available from a commercial marketplace” [27]. We excluded articles that did not involve a mobile phone app (eg, a Web-based or iPad app), medical studies, systematic reviews and meta-analyses, abstracts or congress papers, qualitative studies, study protocols, and studies not including QoL or well-being assessment. We applied no language restrictions.

The search strategy followed the Preferred Reporting Items for Systematic Reviews and Meta-Analysis (PRISMA) guidelines [28]. We searched for trials in The Cochrane Library, Excerpta Medica Database (EMBASE), PsycINFO (via ProQuest), PubMed, Scopus, and MEDLINE (via OvidSP) on July 12, 2017. We extracted trials with the keywords “breast cancer + app,” “breast cancer + mHealth,” “breast cancer + mobile application,” “prostate cancer + app,” “prostate cancer + mHealth,” and “prostate cancer + mobile application” published between January 1, 2000, and July 12, 2017. Two of the authors of this study (ER and EG) independently reviewed the titles and abstracts of the total search yield to identify eligible articles. The full text of the article was retrieved if any reviewer considered a citation potentially relevant. In case of disagreement, a third reviewer (FG) selected the reference finally included, based on inclusion and exclusion criteria. Search results were stored using Endnote version X8 (Clarivate Analytics). Duplications of studies were removed.

#### Data Extraction

Two of the authors of this study (ER and EG) independently reviewed the full text of the articles meeting the eligibility criteria. The interrater agreement (kappa value) was calculated with SPSS version 22 (IBM Corp). The following data were extracted from the selected papers: (1) general patient and study characteristics (year of publication, country of study, language, author affiliations, number of participants, mean age, and cancer type targeted), (2) clinical characteristics (QoL assessment, other variables measured, functionalities, type of validated questionnaire involved and timing for assessment, treatment offered, main clinical results, quality of the study, randomized controlled trial [RCT] design, social media inclusion, theoretical framework based, and quality of the journal), (3) clinical strengths and weaknesses, and (4) patients’ user experience (satisfaction level and comments regarding the apps used). Disagreements were rare and were easily resolved by consensus.

Two of the authors (ORR and ED) independently reviewed the full text of selected articles and extracted the following information: (1) technological characteristics (app name, platform, availability in markets, price, number of downloads, rating, patients targeted, and main features), (2) technological strengths and weaknesses, and (3) patients’ user experience (ratings, health certification obtained, and number of user comments). Disagreements were resolved by a third reviewer (FG). The quality of the included studies was assessed in terms of their design. Nonrandomized, observational, descriptive, and qualitative studies were considered low to medium quality. Quasi-randomized and interventional studies and studies with strongest design were considered of moderate to high quality.

### Reviewing the Apps on the Market

In addition to the systematic review, 3 of the authors (ORR, ED, and CSB) downloaded the apps identified in the studies from the online store. They collected the following information: (1) technological characteristics, (2) technological strengths and weaknesses, and (3) patients’ user experience (satisfaction level and comments regarding the app used). An English language restriction was applied for the mobile phone apps downloaded.

## Results

### General Characteristics

**Figure 1:**
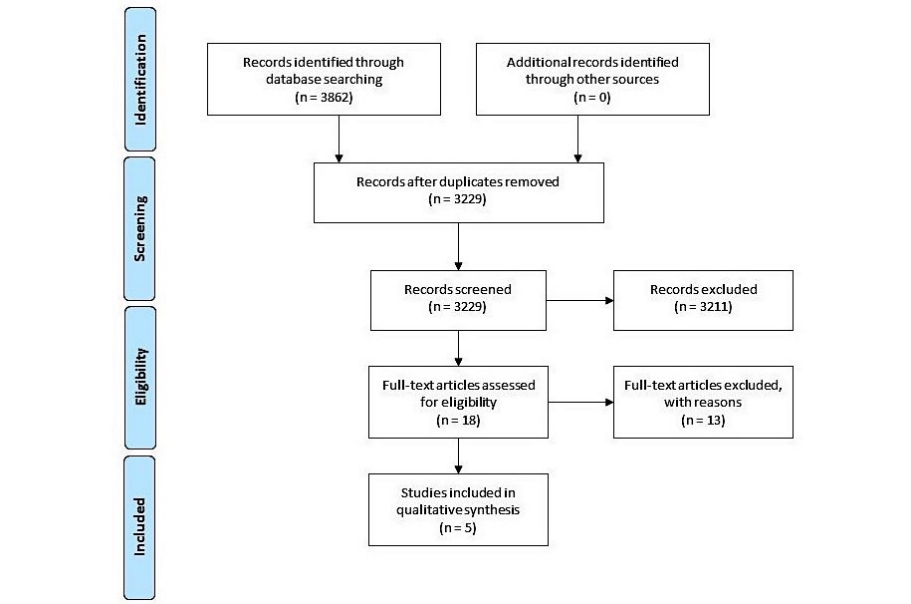
Systematic review of the literature flowchart.

The search of the electronic databases retrieved 3862 citations. After removal of duplicates, 3229 remained and were evaluated on the basis of title and abstract. Of these, 3211 were discarded because they clearly did not meet the inclusion criteria. Based on titles and abstracts, 18 records were selected for full text screening; 13 out of these 18 [29-41] being discarded for various reasons (see Multimedia Appendix 1). A total of 5 publications [42-46] were finally included. An interrater agreement of kappa=.561 was found, reaching a moderate agreement according to Landis and Koch [47]. All chosen studies were deemed to be of sufficient quality to contribute equally to the thematic synthesis. A PRISMA flowchart is shown in Figure 1 [28].

The 5 studies included a total of 644 patients, mean age 52.16 years (sample sizes and mean ages listed in Table 1). Of these, 3 studies were conducted in Korea [42,44,46], 1 in the United States [43], and 1 in Sweden [45]. All main authors affiliations were university departments [42-46]. The majority of the studies targeted breast cancer patients [42-44,46]; only 1 focused on prostate cancer patients [45]. Other general characteristics of the studies included are summarized in Table 1.

### Clinical and Technological Characteristics

Regarding the clinical approach, 4 of the 5 included studies referred to apps that assessed QoL [43-46]. Among the other variables measured were depression status (mood, anxiety, and sleep satisfaction) [42]; daily food intake, daily exercise, daily body weight, weight efficacy, anthropometrics, and physical activity [43]; sleep disturbance [44]; sense of coherence, bladder and bowel function, fatigue, pain, anxiety, distress, sleep, and flushing [45]; physical activity [43,46]; and user satisfaction [46].

All studies allowed patients to collect patient-reported outcome measures [42-46], and 3 included a related-intervention app [43,45,46].

**Table 1:** General characteristics of included studies (n=5).

Study	Publication year	Country/language	Participant number	Mean age	Cancer type
Kim et al [42]	2016	Korea/Korean	78	44.35	Breast
McCarroll et al [43]	2015	United States/English	50	58.4	Breast
Min et al [44]	2014	Korea/Korean	30	45	Breast
Sundberg et al [45]	2017	Sweden/Swedish	130	69	Prostate
Uhm et al [46]	2017	Korea/Korean	356	50.3	Breast

The included studies measured QoL through different questionnaires such as the Functional Assessment of Cancer Therapy–General (FACT-G) [48], a generic core questionnaire that comprises 27 items divided into 4 domains (physical, functional, emotional, and social well-being) [49]; the EuroQol 5 Dimensional Questionnaire (EQ-5D-3L) [50], a generic health outcome instrument comprising 5 dimensions (mobility, self-care, usual activities, pain/discomfort, and anxiety/depression) [49]; the European Organization for Research and Treatment of Cancer Quality of Life Questionnaire–Core (EORTC QLQ-C30) [51], a 30-item generic cancer questionnaire that consists of 5 function scales (physical, role, emotional, cognitive, and social), a global health scale, 3 multi-item symptom scales (fatigue, nausea/vomiting, and pain), and 6 single item scales (dyspnea, sleep, appetite, constipation, diarrhea, and financial difficulties due to disease) [49]; and the EORTC complementary modules on prostate cancer, QLQ-PR25 [52], and breast cancer, QLQ-BR23 [53]. One study did not assess QoL symptoms [42], focusing only on well-being assessment through the Patient Health Questionnaire (PHQ-9) [54], which measures both the presence and the severity of 9 depression symptoms and is able to yield a diagnosis [55]. Only one of the included studies reported the theoretical framework on which the app relied. Further details on other clinical variables assessed by the included studies are reported in Table 2.

Concerning the main clinical results, the adherence to the self-reporting measures was associated with higher accuracy of depression screening [42]. Moreover, the compliance with the daily self-reporting rates was not affected by depression symptoms or health-related quality of life (HRQoL) status reported by the patients [44]. Of the 3 studies that included intervention [43,45,46], only 2 reported a QoL improvement [45,46]. The real-time assessment and management of symptoms through Interaktor [45] produced significantly less burden in emotional functioning, insomnia, and urinary-related symptoms at T2 (after end of treatment, which ranged from 5 to 8 weeks) and at T3 (3 months after end of treatment) compared with the control group. Likewise, the 12-week regimen of aerobic and resistance exercise through Smart After Care [46] produced a significant improvement in physical function, physical activity, and QoL at baseline and 12 weeks. The 4-week comprehensive lifestyle program focusing on nutrition quality, physical activity, and improving eating displayed by LoseIt! [43] did not affect QoL.

With regard to the quality of the studies, 3 were considered of low-to-medium level [42,43,44], and 2 a medium-to-high level of quality [45,46].

The study design involved 2 prospective, nonrandomized multicenter controlled trials [45,46], 1 with control group [45], but no RCT designs were included. None of the 5 studies involved social media features, and only 1 study [43] mentioned the theoretical framework, social cognitive theory, on which it was based.

Concerning the publication journals, 4 out of the 5 included studies [42,43,45,46] were published in the last 3 years (2015-2017). Scientific journals are ranked yearly based on impact factor data, and the Journal Citation Reports (JCR) published by Clarivate Analytics are widely used as a quality indicator. The JCR ranks journals into categories based on which quartile of the impact factor distribution the journal occupies for that category: Q1 represents the top 25% of journals in the distribution, Q2 between the top 50% and top 25% of journals, and Q3 between the top 75% and top 50% of journals. According to the 2017 JCR, 4 studies [42-44,46] were issued in journals ranking Q1 or Q2.

With regard to technological characteristics, all of the 5 studies included provided the names of the mobile phone apps evaluated [42-46]. There were 2 studies involving the same app [42,44] (see Table 3). The majority of the studies included an app targeted at cancer patients [42,44-46]. The main features of the apps were focused on exercise and nutrition logging [43]; collection of patient-reported outcomes [44]; early detection, reporting, and management of symptoms [45]; and exercise by a step counter [46]. More technological characteristics can be seen in Table 3.

App functionalities included the following: (1) customization and personalization features (create a menu plan and calorie tracker [43] to establish an individual profile), (2) motivational features (providing feedback), and (3) social features (developing a community for inspiration).

**Table 2:** Clinical characteristics of included studies (n=5).

Study	QoL^a^ assessment	Functionalities	Validated questionnaire/timing	Treatment offered	Quality of study
Kim et al [42]	No	PRO^b^: daily mental health ratings over a 48-week period	PHQ-9^c^ via app biweekly	No	Low-medium
McCarroll et al [43]	Yes	PRO: daily, real-time, and motivational feedback + intervention	FACT-G^d^, WEL^e^ at baseline and at 4-week follow-up	Comprehensive lifestyle program	Low-medium
Min et al [44]	Yes	PRO: daily basis over a 90-day period	BDI^f^, EQ-5D-3L^g^ via app on a daily basis for 90 days	No	Low-medium
Sundberg et al [45]	Yes	PRO: daily, real-time assessment of symptoms and concerns during radiotherapy + intervention^h^	EORTC QLQ-C30^i^, EORTC QLQ-PR25^j^ via app daily at any time during radiotherapy and 3 weeks after completion	Management of symptoms	Medium-high
Uhm et al [46]	Yes	PRO + intervention^h^	EORTC QLQ-C30, EORTC QLQ-BR23^k^ at baseline and 12 weeks	12-week regimen of aerobics	Medium-high

^a^QoL: quality of life.

^b^PRO: patient-reported outcome measures.

^c^PHQ-9: Patient Health Questionnaire–9.

^d^FACT-G: Functional Assessment of Cancer Therapy–General.

^e^WEL: Weight Efficacy Lifestyle questionnaire.

^f^BDI: Beck Depression Inventory.

^g^EQ-5D-3L: EuroQol 5-Dimension 3-Level survey.

^h^Significant improvement in quality of life.

^i^EORTC QLQ-C30: European Organization for Research and Treatment of Cancer Quality of Life Questionnaire–Core.

^j^EORTC QLQ-PR25: European Organization for Research and Treatment of Cancer Quality of Life Questionnaire–Prostate.

^k^EORTC QLQ-BR23: European Organization for Research and Treatment of Cancer Quality of Life Questionnaire–Breast Cancer.

**Table 3:** Technological characteristics of included studies (n=5).

Study	App name	Platform	Available in markets	Price	Downloads	Ratings	Patients Targeted
Kim et al [42]	Pit-a-Pat	Android/iOS	No	Unknown	Unknown	Unknown	Yes
McCarroll et al [43]	LoseIt!	Android/iOS	Yes	free/premium	Android: 5,000,000-10,000,000	Android: 4.4; iOS: 4.0	No
Min et al [44]	Pit-a-Pat	Android/iOS	No	Unknown	Unknown	Unknown	Yes
Sundberg et al [45]	Interaktor	Unknown	No	Unknown	Unknown	Unknown	Yes
Uhm et al [46]	Smart After Care	iOS	No	Unknown	Unknown	Unknown	Yes

### Clinical and Technological Strengths and Weaknesses

Regarding the studies’ strengths, 2 of them involved the same app called Pit-a-Pat [42,44]. Pit-a-Pat was developed for cancer patients to self-report factors related with the diagnosis itself and the subsequent treatments: (1) sleep-disturbance symptoms, (2) acute symptoms related to cytotoxic chemotherapeutic agents, and (3) medication diary for antihormonal treatment. Kim et al [42] studied the accuracy of a mobile mental health tracker for depression screening, as well as the adherence on screening accuracy. For that purpose, daily patient reports of anxiety symptoms, mood, and sleep satisfaction were collected through a reliable and valid questionnaire (PHQ-9). The app involved user-friendly functionalities in the form of a facial emoticon scale. Min et al [44] studied the patient’s self-reported sleep disturbance, HRQoL status, and depression symptoms via the Pit-a-Pat app on a daily basis for 90 days with standardized questionnaires. Push notifications were sent to participants daily at 9 AM and 7 PM.

LoseIt! [43] was a Web- and mobile-based app, not cancer targeted, used for logging food intake and volitional exercise. McCarroll et al [43] aimed to assess a 4-week comprehensive lifestyle program with emphasis on nutrition quality, physical activity, and improving eating self-efficacy delivered using a beta health care provider version of LoseIt! in which the patients could log daily food choices, daily exercise type and duration, and daily body weight over the treatment period. Participants received motivational patient-provider feedback notifications (phone call, email message, and/or a push notification) in response to their individual input in the LoseIt! app.

Interaktor [45] was codesigned by patients and health care personnel as an interactive app for mobile phones and tablets. Interaktor was specifically intended for early detection, reporting, and management of symptoms and concerns during treatment for prostate cancer. Daily reports via the app enable instant support from a nurse in early detection and management of symptoms and concerns in real-time during treatment for prostate cancer. The app features included symptom assessment, a risk assessment model for alerts directly to a nurse, continuous access to evidence-based self-care advice, and links to relevant websites. The apps sent patients a reminder message if they had not submitted their report. In addition, the system sent 2 alerts (yellow and red) to the patients, depending on their symptom occurrence and frequency. The red meant a higher priority (should contact the nurse within an hour), and the yellow alert indicated that the nurse should be called that day. Sundberg et al [45] included a control and an intervention group in their study, which used the app for daily, real-time assessment and management of symptoms and concerns during radiotherapy treatment. Participants were asked to send reports daily and at any time point when they felt unwell during the radiation treatment (5 to 8 weeks) and the following 3 weeks.

Smart After Care [46] was a newly developed mobile phone exercise app. This app recorded minutes of physical activity weekly and established a weekly goal for minutes of activity beginning in the second week. Every week, the achievement rate was displayed by the app. Also, patients receiving hormonal therapy could watch a video clip of resistance and stretching exercises through the app. This study included standardized QoL questionnaires and a user satisfaction survey in the intervention group. Patients in the study by Uhm et al [46] performed a 12-week regimen of aerobic and resistance exercise through Smart After Care, where the intervention group received a pedometer and Smart After Care to perform 150 or 90 minutes of aerobic exercise.

Among the main weaknesses could be cited the small samples of the studies [42-44] and the lack of RCT protocols and framework-based apps. Moreover, we can report only 1 app for free download on the market [43], which was used with breast cancer patients despite not being cancer-focused.

### Patients’ User Experience

Only 1 study [46] reported information regarding patient satisfaction level, and only 1 app showed a quality certification [43] and a considerable number of user comments (see Table 4). The mean Likert scale response for overall patient satisfaction with the service was 4.27/5 in the mHealth group [46].

**Table 4:** Patient satisfaction levels provided by the health literature review and the online store search (n=5).

Study	Patients satisfaction	Ratings (OSR^a^)	Health certification (OSR)	Number of user comments (OSR)
Kim et al [42]	Unknown	Unknown	Unknown	Unknown
McCarroll et al [43]	Unknown	Android: 4.4; iOS: 4.0	Helix’s CLIA^b^ certified and CAP^c^ accredited lab	Android: 61,063; iOS: 374,815
Min et al [44]	Unknown	Unknown	Unknown	Unknown
Sundberg et al [45]	Unknown	Unknown	Unknown	Unknown
Uhm et al [46]	Satisfied with use	Unknown	Unknown	Unknown

^a^OSR: Online store research.

^b^CLIA: Certified Laboratory Improvement Amendments.

^c^CAP: College of American Pathologists.

## Discussion

### Overview

The use of mobile phone apps for health purposes continues to increase [7], and currently thousands and thousands of health apps are available on the online market. They target different health conditions, including cancer. Health apps represent an opportunity to monitor psychological distress and QoL related to cancer and its associated treatments. Our systematic review shows that the scientific literature referring to apps targeting breast or prostate cancer patients and involving QoL and/or well-being measurements is very modest, as we only could identify 5 studies meeting the inclusion criteria. However, the quality and dates of publication show a current scientific interest in this research topic.

The most recent reviews involving focused cancer apps started the searching methodology by looking for apps on the online stores, followed by searching bibliographic databases of health literature [8,13]. However, we considered it more appropriate to start by determining whether rigorous trials had been published on cancer-focused apps. Hence, we conducted the systematic literature review first, and then we downloaded the apps from the market stores to examine them.

### More Evidence-Based Apps Are Needed

Despite the increase in the number of health care apps available [15], only a very few of them discussed in the scientific literature focus on QoL and/or well-being assessment in breast or prostate cancer patient even though breast and prostate cancer are the most prevalent cancers diagnosed [1] and QoL and well-being are frequently assessed to determine the health status of cancer patients [2,3]. There are only 2 studies that reported QoL improvement by using related-treatment health apps [45,46].

Related research on health apps for cancer patients was identified but not included in the review due to the following reasons (see Multimedia Appendix 1): it did not involve mobile phone apps [30-32,35,37,38], it did not assess QoL or well-being [29,33,34,36,40], or it involved qualitative studies focused on feasibility or patient opinions [39,41].

We selected the QoL measure considering it has a wide range of variables involving other psychological measures (eg, cognitive, emotional, and social abilities) and not only as performance status and daily functional activities [29], symptom experience [33], or chemotherapy-related symptoms (nausea, vomiting, fatigue, mucositis, hand-foot syndrome, and diarrhea) [34].

We have defined well-being as existing levels of general anxiety and depression symptoms and not only as perceived stress level [36] assessed by the Perceived Stress Scale (PSS) [56], a scale “designed to measure the degree to which situations in one’s life are appraised as stressful” and suggested by its own authors “as an outcome measure of experienced levels of stress” [56], not well-being levels.

In our review, only 1 of the selected papers provided information regarding patient satisfaction level [46], therefore it is not possible to draw conclusions about the patient satisfaction or perceived effectiveness of the current apps.

Most of the apps referred to in the scientific literature targeted breast cancer, as in previous reviews [8,13,16-18], and only 1 study focused on prostate cancer. All of these health apps were developed by university institutions; in contrast with the review of Mobasheri et al [18], which reports that a minority of medical professionals were involved in the apps. Our results showed that, a priori, all the studies have been hosted by significant research and educational institutions.

With regard to the technological characteristics, it should be noted that the 3 apps specifically designed for cancer patients [42,44-46] were not available for download on the market. Furthermore, only 1 [43] out of 4 total apps included was available for download at the online store, and despite this app (LoseIt!) not being specifically patient-targeted, it was used by 50 breast cancer patients to manage exercise and nutrition concerns.

### Clinical and Technological Strengths and Weaknesses

Regarding the studies’ strengths, the use of related-treatment mobile phone apps has resulted in a significant improvement in cancer patients’ QoL [45,46]. Some features like displaying daily patient reports in real time and providing personalized feedback [43,45] have also been pointed out as a significant advantage of the apps [34]. Moreover, if the assessment involves user-friendly functionalities such as a facial emoticon scale [42], which could be adapted to the small phone screen, this may facilitate user participation, potentially making the data more useful. Previous studies [31] have reported important usability adaptation, incorporating several design decisions to account for patients with various disabilities (eg, impaired vision), presenting only 1 question at a time to patients. The easy and visual (similar to a stock chart) way of obtaining the information displayed by the app as feedback on the symptoms report form at any time during the study has also been mentioned as a relevant strength [29]. Also, it is notable that when some researchers wished to test a general-population–targeted app with cancer patients, they delivered a cancer-focused health care provider beta version [43] and designed the user interface of the app based on reliable guidelines developed by the National Cancer Institute, like previous authors did [31].

Participants using their own mobile phones have mentioned this as better than being provided with an additional device [44], probably because in the latter case they must deal with 2 mobile phones in their daily life or because they prefer some relative freedom for testing the app, meaning the possibility of using the app at their convenience with no minimum amount of time to be spent using the app, as previous authors have reported [41]. In contrast, other studies have pointed out patient preferences for using a device without phone functionality [57] instead of using their own phones. Users strongly appreciate the use of no personal patient identifiers or other information stored on the devices used [30], as well as pseudonymizing and data protection [4]. Apps that can be used on more than 1 device could provide the patients with more possibilities to test them, such as LoseIt! [43], which offers both website and mobile versions for users, or Interaktor [45], available for mobile phones and tablets. The real-time feedback component and the motivational feedback notification are considered relevant strengths as well. The flexibility in the self-reporting task [45] could probably be a more suitable option in oncological settings than prefixed hours of a day [44] because of the patients’ highly variable functional status during the day, largely dependent on the medical treatments.

Among the main weaknesses could be (1) no cancer-focused apps are being used in studies involving cancer patients [36,40,41,43,57], (2) many of these studies have small samples, (3) studies are without rigorous design based on RCTs, (4) studies are not free to the user, (5) no theoretical framework is reported, and (6) there are usability and accessibility issues with cancer patients.

It is important to highlight the relevance of using cancer-focused apps in oncological settings, as cancer patients could be considered vulnerable recipients [4]. People suffering from cancer have to struggle with quick relapses, bad prognoses [4], side effects caused by cancer treatments, and psychological distress [2]. Also, they represent a population interested in doing everything possible to improve their health [4], so they could be interested in using apps that do not constitute reliable and accurate tools for them, which is even worse if the patients have to pay for them. Mobasheri et al [18] reported that of the 30 apps reviewed, which functioned as self-assessment tools for breast disease, only 2 (2/30, 7%) had a documented evidence base (the rest relied on empirical data). It is imperative to develop apps and other health information and technology systems specifically targeted to cancer patients.

Although some encouraging results have been reported using apps in cancer patients [34], bigger samples sizes and framework-based and RCT designs are needed in order to obtain stronger research conclusions. Otherwise, serious concerns could arise regarding the lack of validation [12] and quality control [15] of the studies. Furthermore, the identification of rigorous trials involving empirical testing of these mobile phone apps in oncological settings is imperative, as none of the selected studies in this review followed a randomized method and only 1 was based on a previous framework. The use of theories, models, and frameworks for apps will help to identify the mechanisms, approaches, and functionalities that work best.

Cancer patients and survivors could have cognitive deterioration due to treatments. Therefore, usability and accessibility are relevant aspects to be considered in the development process of these apps, especially when they are intended for older people. Apps not designed for cancer patients and survivors could entail difficult challenges for them, resulting in reduced adoption and engagement rates. Equally, the large variety of apps available makes it difficult to establish which of them are the most adequate for breast and prostate cancer patients and what is the best way to use them. Also, patients could become overwhelmed due to the huge number of cancer apps available [11]. Because of this, stronger efforts should be made to consolidate the evidence base, effectiveness, and safety of cancer-focused apps [8].

In line with previous research [8,13,16-18,20,21], we consider a main challenge the task of ensuring that those apps that are planned to be used with cancer patients be effectively cancer-focused, meaning that they should have been originally designed for, tested on, and adapted to the cancer population. Only if this technology is evidence-based and targeted to cancer patients can health care providers guarantee the apps’ safety, accuracy, reliability, and high quality and be able to recommend them in oncological care settings.

### Patients’ Satisfaction With the Health Apps

Regarding patients’ satisfaction, it is noteworthy that only 1 app of the 3 reviewed reported a quality certification and showed user comments regarding its use. Moreover, it is relevant that this app was not cancer-focused and was the only one available for download at the online store. Only user-friendly and quality-certified apps should be provided to cancer patients. Thus, these health apps must be available for download at market stores once they are certified as useful tools for cancer patients. It would probably be helpful as well that these apps provide new users with comments about other patient’s experiences, in order to obtain a more powerful overview of the main features included in the app.

More evidence on the patient satisfaction level using health apps for QoL and/or well-being assessment in oncological settings is needed. In our review, only 1 study [46] focusing on cancer patients provided information about satisfaction level.

People affected by cancer are usually open to strategies that could have a positive influence on their disease [4]. Probably due to this fact, mobile phone developers and health care teams involved in oncological settings should be especially careful with the apps that are going to be used and tested by this population and implement patient-centered design approaches. Current RCTs are still being developed that might produce promising data to help reach a high-quality evidence base for apps for cancer patients’ use [32,57-59].

### Limitations

Our study had certain limitations. Our selection criteria intentionally excluded apps that were not specifically focused on breast or prostate cancer patients. We considered only the assessment of 2 main psychological variables in psycho-oncological care: QoL and well-being (anxiety and depression symptoms). Additional studies could consider other psychological measures such as fatigue or the secondary symptoms produced by the cancer treatments. Although our data search represents a wide range of peer-reviewed journals, we might have missed studies that were not identified with our search terms or that were not published.

### Conclusions

Despite the existence of hundreds of studies involving mobile phone health apps used by cancer patients, there is a lack of rigorous trials regarding QoL and/or well-being assessment in breast and/or prostate cancer patients. More evidence-based apps, which could be tested in future RCT protocols, are still needed. However, promising results are expected to be available from some RCTs that are still running. A strong and collective effort should be made by all health care providers to determine those cancer-focused apps that provide useful and reliable tools for cancer patients’ disease management.

## Appendix

Multimedia Appendix 1 Reasons for exclusion of studies.

Multimedia Appendix 2 Overview of the systematic review.

## Abbreviations

BDI: Beck Depression Inventory
CAP: College of American Pathologists
CLIA: Clinical Laboratory Improvement Amendments
EORTC QLQ-C30: European Organization for Research and Treatment of Cancer Quality of Life Questionnaire–Core
EORTC QLQ-BR23: European Organization for Research and Treatment of Cancer Quality of Life Questionnaire complementary module for breast cancer patients
EORTC QLQ-PR25: European Organization for Research and Treatment of Cancer Quality of Life Questionnaire complementary module for prostate cancer patients
EQ-5D-3L: EuroQol 5-Dimension questionnaire
FACT-G: Functional Assessment of Cancer Therapy–General
HRQoL: health-related quality of life
mHealth: mobile health
PHQ-9: Patient Health Questionnaire–9
PRISMA: Preferred Reporting Items for Systematic Reviews and Meta-Analyses
PRO: patient-reported outcome measures
PSS: Perceived Stress Scale
QoL: quality of life
RCT: randomized controlled trial
OSR: online store research
WEL: Weight Efficacy Lifestyle questionnaire
