# Supplementary material for: Mobile Phone Apps for Quality of Life and Well-Being Assessment in Breast and Prostate Cancer Patients: Systematic Review
Source: JMIR Mhealth Uhealth. 2017 Dec 4;5(12):e187. doi: 10.2196/mhealth.8741 (PMC5735250; doi:10.2196/mhealth.8741)
Supplement: Multimedia Appendix 1 [file mhealth_v5i12e187_app1.pdf]

Multimedia Appendix 1. Reasons for studies exclusion.

Study	Reason for exclusion
Egbring et al. [29]	No QoL assessment.<br>Common Terminology Criteria for Adverse Events (CTCAE) v4.0, Eastern Cooperative Oncology Group (ECOG) Performance Status via app.
Foley et al. [30]	No mobile phone app. It is an iPad app.
Fromme et al. [31]	No mobile phone app. It is a touch screen with rotation ability from laptop mode to tablet mode.
Fu et al. [32]	No mobile phone app. It is a website accessible through a mobile phone.
Hyun-Ju & Bok-Hee [33]	No QoL assessment.<br>Diet, physical exercise, and pain by using the mobile phone app.<br>Symptom Experience Scale (SES), social support, and<br>Mishel Uncertainty in Illness scale (MUIS) by using self-reporting questionnaires.
Kearney et al. [34]	No QoL assessment.<br>Chemotherapy Symptom Assessment Scale (C-SAS).
Matthew et al. [35]	No mobile phone app. It is a PDA app.
Quintiliani et al. [36]	No QoL assessment.<br>Perceived Stress Scale (PSS) assessment.
Skolarus et al. [37]	No mobile phone app. Telephone assessment with interactive voice responses.
Somers et al. [38]	No mobile phone app. Live video-conferencing using a tablet.
Sundberg et al. [39]	Qualitative Study.
Valle et al. [40]	Anxiety assessment but only regarding weight gain.
Young-Afat et al. [41]	Qualitative Study.
