# Supplementary material for: Mobile Phone Apps for Quality of Life and Well-Being Assessment in Breast and Prostate Cancer Patients: Systematic Review
Source: JMIR Mhealth Uhealth. 2017 Dec 4;5(12):e187. doi: 10.2196/mhealth.8741 (PMC5735250; doi:10.2196/mhealth.8741)
Supplement: Multimedia Appendix 2 [file mhealth_v5i12e187_app2.pdf]

This is a Multimedia Appendix to a full manuscript published in the JMIR mHealth and uHealth. For full copyright and citation information see http://dx.doi.org/10.2196/mhealth.8741

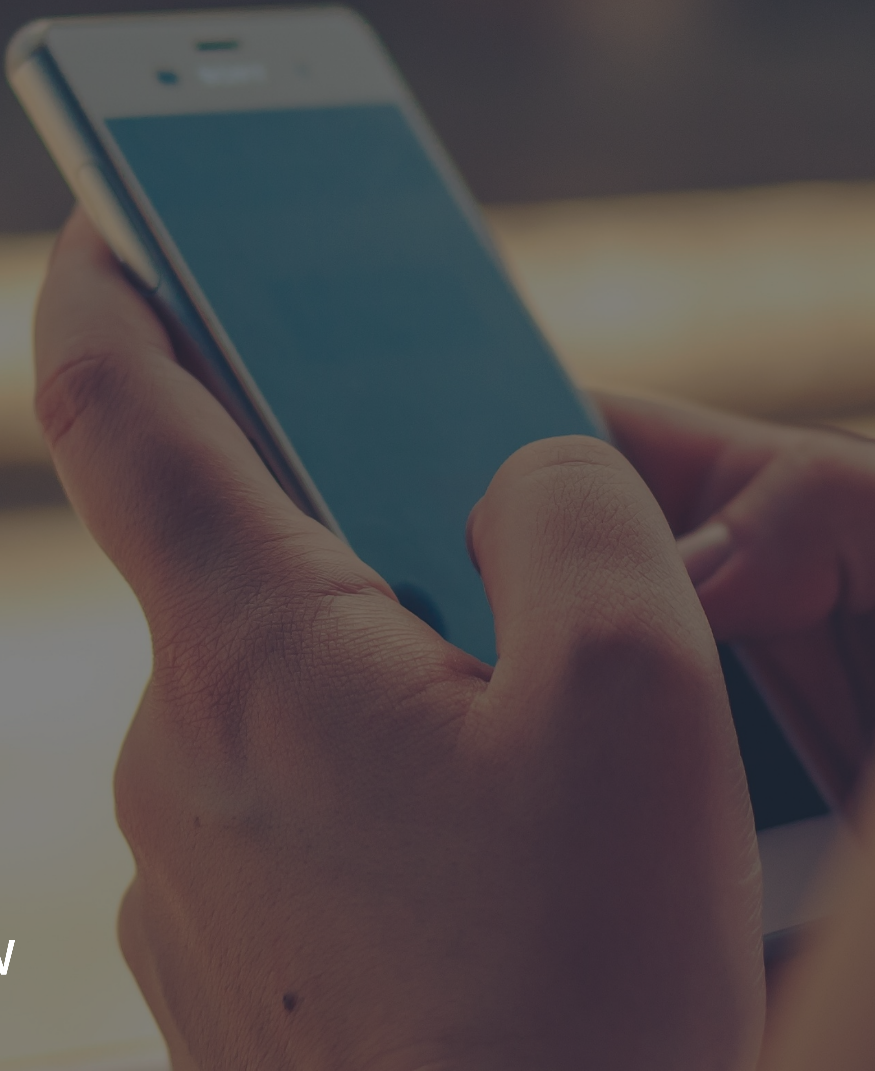

Mobile Phone Apps

for Quality of Life and Well-being Assessment in Breast and Prostate Cancer Patients: Systematic Review

doi:10.2196/mhealth.8741

Authors:
Esther Rincon, Ph.D.;
Francisco Monteiro-Guerra, MS;
Octavio Rivera-Romero, Ph.D.;
Enrique Dorronzoro-Zubiete, Ph.D.;
Carlos Luis Sanchez-Bocanegra, Ph.D.;
Elia Gabarron, Ph.D.

Editor:
Gunther Eysenbach

INTRODUCTION

- The number of new cancer cases diagnosed every year worldwide is rapidly rising:
  14.1 M in **2012** to over **20 M** predicted by **2030**
- Breast and prostate cancers are the most prevalent diagnosed in women and men, respectively
- **30% to 40%** of cancer patients suffer from psychological distress – anxiety and depression
- This associates with a poorer quality of life (QoL)

- Mobile phone health apps are increasingly gaining attention in oncologic care
- Useful for monitoring patients and provide valuable data for both patients and healthcare professionals
- These apps have the potential to empower cancer patients and improve their QoL and well-being
- The number of studies concerning the use of these technologies to support breast and prostate cancer patients is rising

---

- However, there are only a few apps that are designed for these individuals
- There are still important concerns regarding the quality of available apps and satisfaction of use

There is a need to properly review mobile health apps focused on QoL and well-being in breast and prostate cancer patients

GOAL

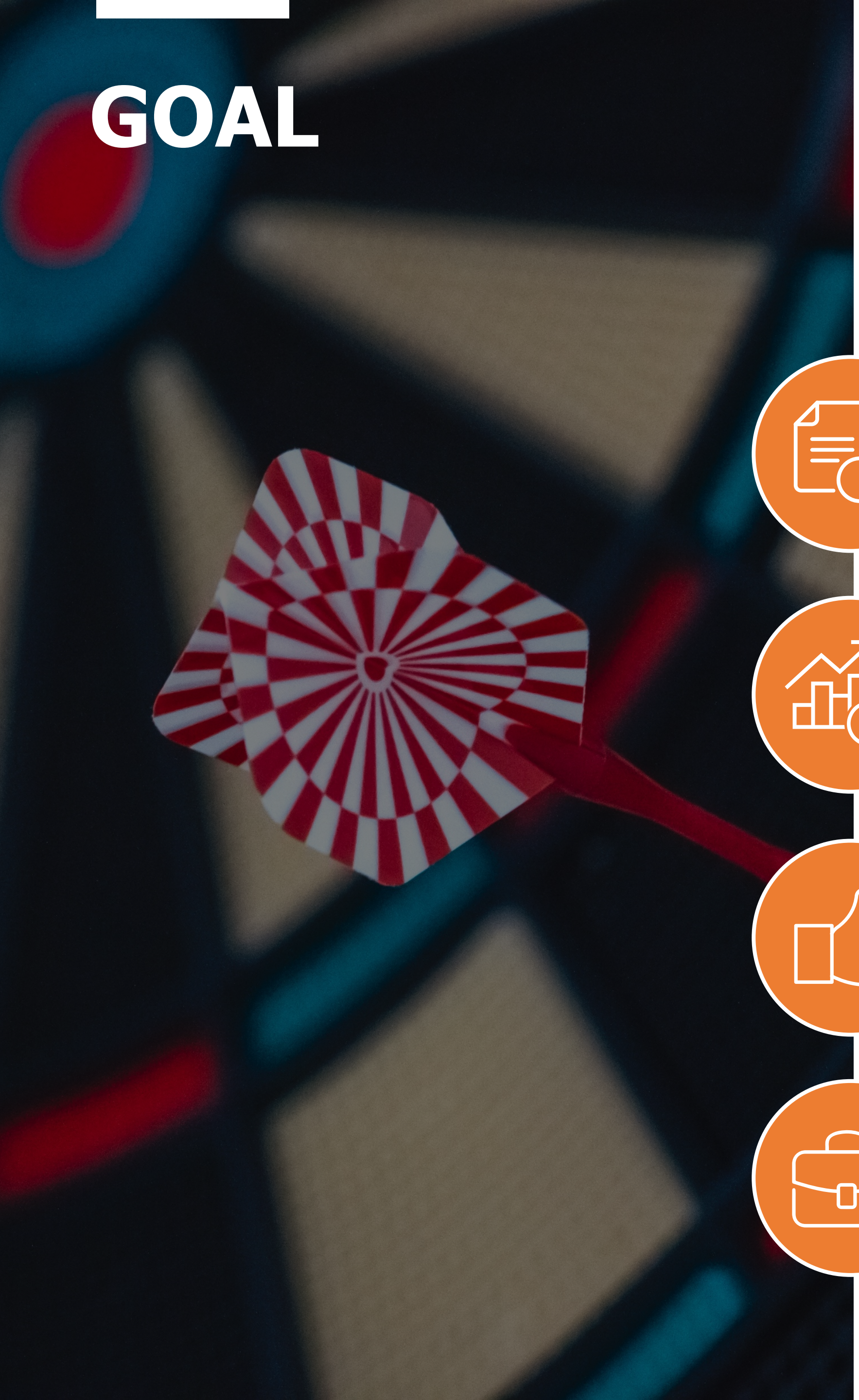

The objectives of this study

- To identify evidence-based mobile phone health apps focused on QoL and well-being (anxiety and depression symptoms) and targeting breast and/or prostate cancer patients
- To recognize their clinical and technological characteristics
- To categorize their clinical and technological strengths and weaknesses
- To determine patients' user experience

METHODS LITERATURE REVIEW

Selection Criteria

- Trials; peer-reviewed studies; published between January 1, 2000 and July 12, 2017
- Studies including a mobile phone app focused on QoL and/or well-being and used by breast and/or prostate cancer patients
- Excluded articles: not involving a mobile phone app; medical studies; systematic reviews and meta-analyses; abstract or congress papers; qualitative studies; study protocols; and studies not including QoL or well-being assessment
- No language restrictions were applied

Search strategy

- PRISMA guidelines
- Search done on July 12, 2017
- Extracted trials from: the Cochrane Library; EMBASE; PsycINFO (via ProQuest); PubMed; Scopus; and MEDLINE (via OvidSP)
- Keywords: “breast cancer + app”; “breast cancer + mHealth”; “breast cancer + mobile application”; “prostate cancer + app”; “prostate cancer + mHealth”; and “prostate cancer + mobile application”

Note: The systematic research protocol is registered at PROSPERO [CRD42017073069]

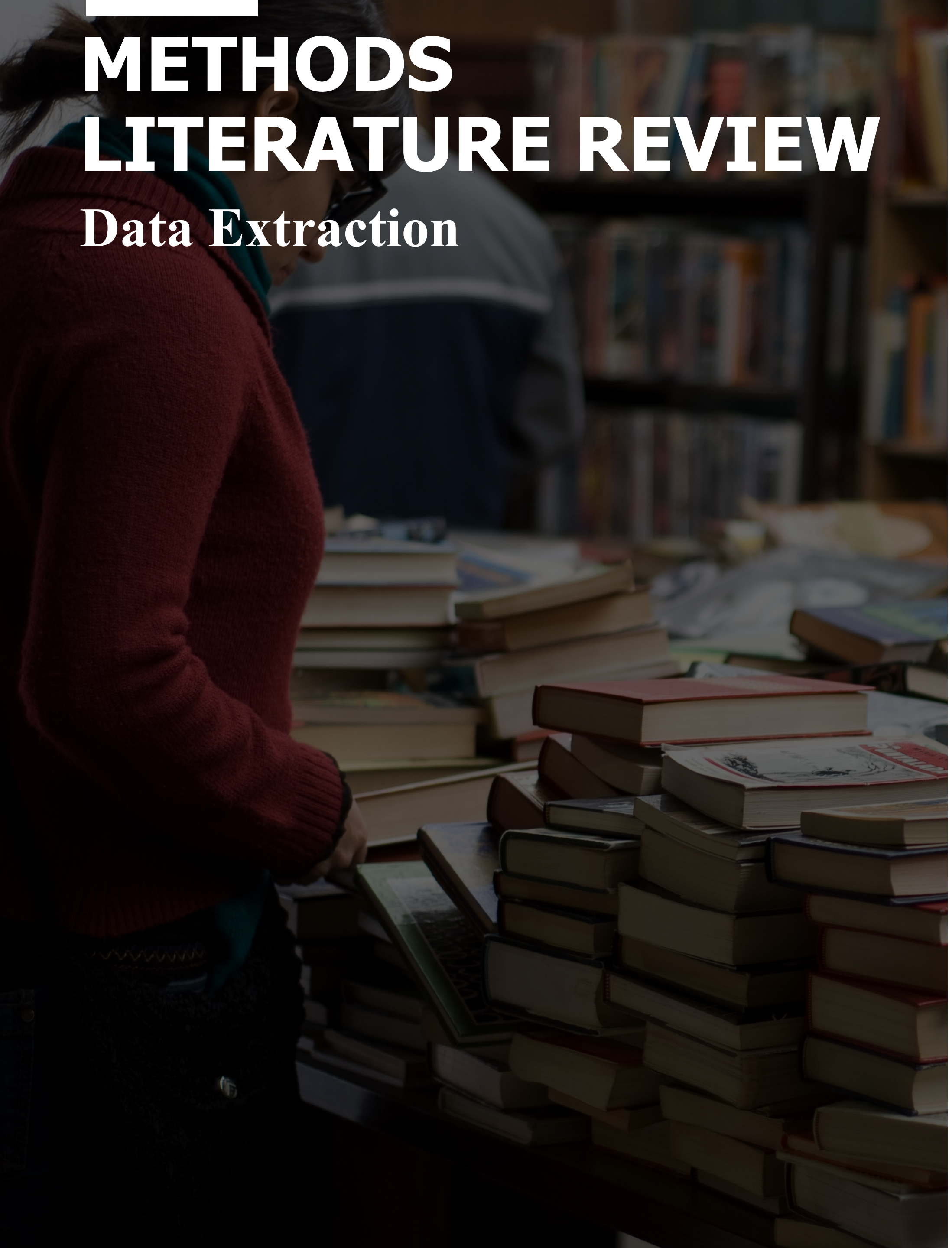

METHODS LITERATURE REVIEW

Data Extraction

General patient and study characteristics

Clinical characteristics

Clinical strengths and weaknesses

Technological characteristics *

Technological strengths and weaknesses *

Patients’ user experience *

* Information complemented with market review of apps identified

METHODS MARKET REVIEW

Data Extraction

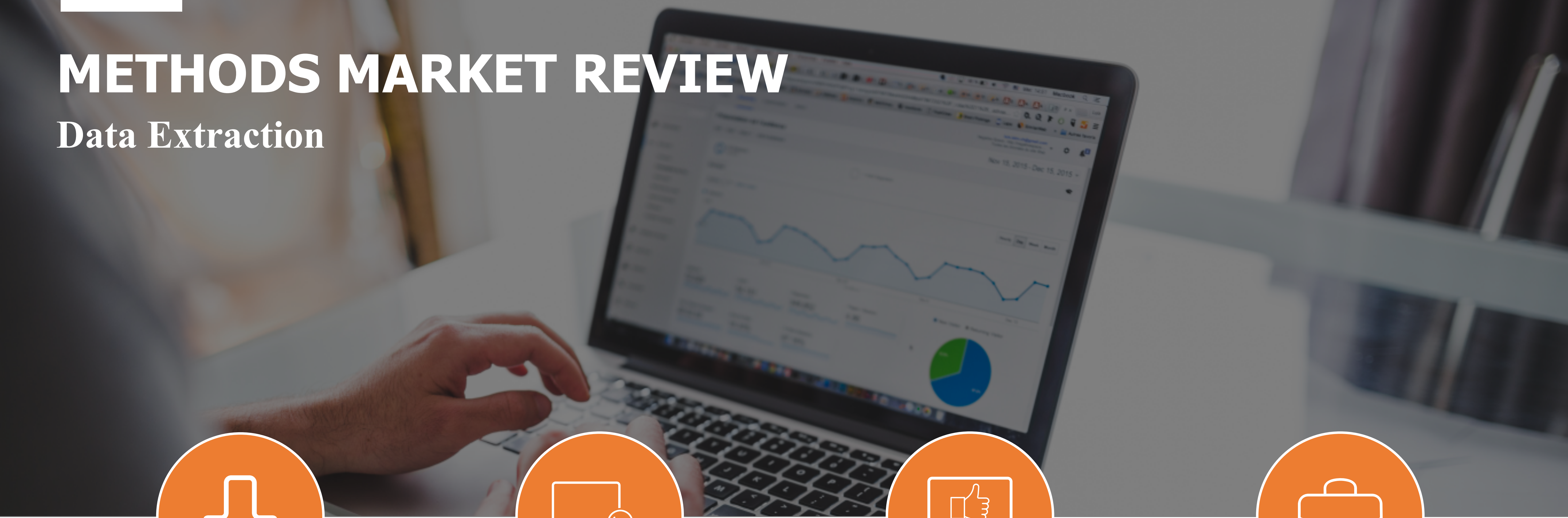

Mobile phone apps identified in literature downloaded from online store

Further detailing technological characteristics

Further examining technological strengths and weaknesses

Identifying user experience (satisfaction level and comments regarding the app used)

RESULTS DATA BASE SEARCH

Based on titles and abstracts, 18 records were selected for full text screening

5 publications were finally included among the three reviewers (ER, EG and FG)

Inter-rater agreement of kappa was found in the first review round (kappa=.561)

All the chosen studies were deemed to be of sufficient quality to contribute equally to the thematic synthesis

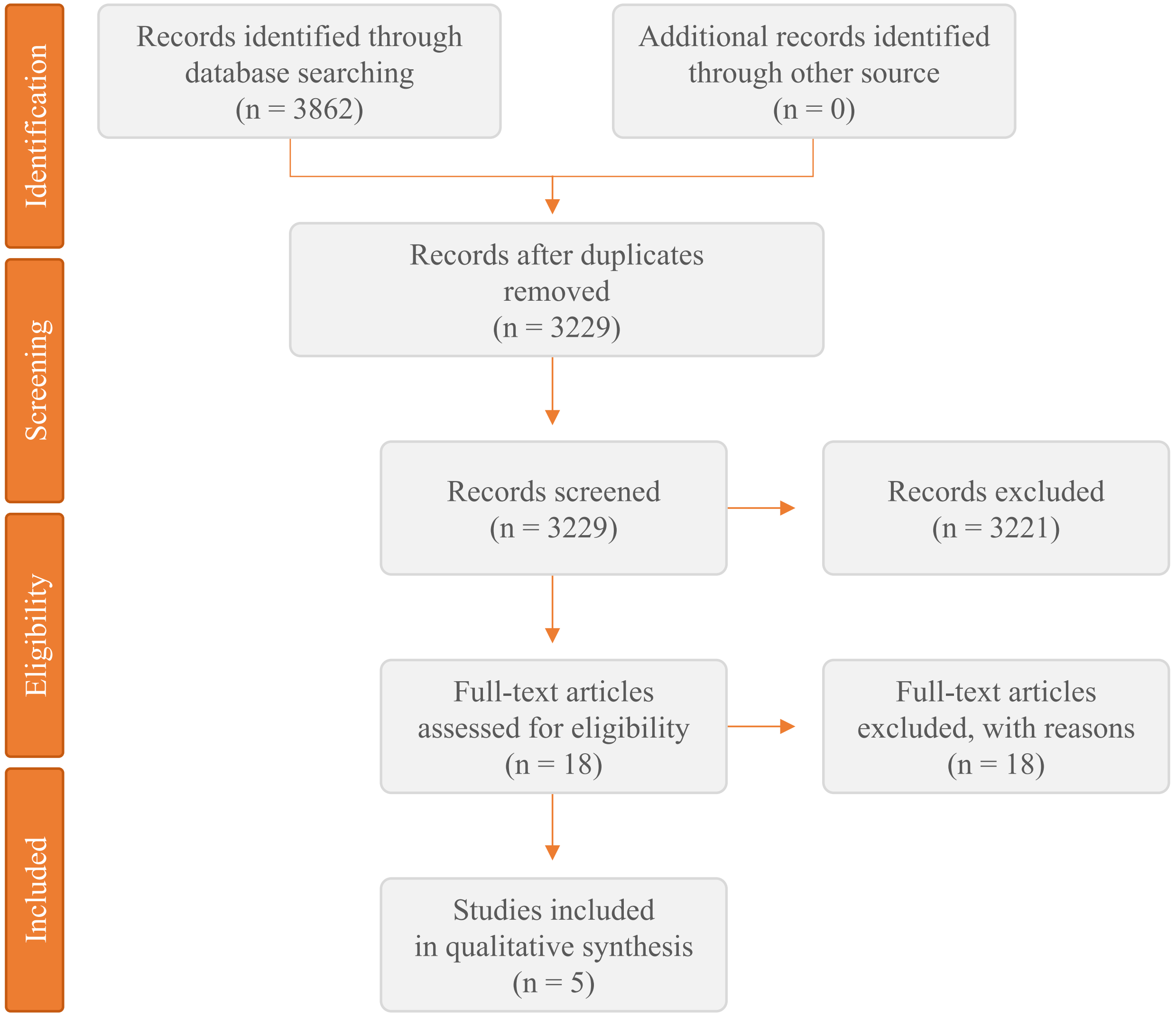

RESULTS GENERAL CHARACTERISTICS

There were 5 studies included, with a total of 644 patients, mean age 52.16 years

3 studies were conducted in Korea, 1 in the United States and 1 in Sweden

The majority of the studies targeted breast cancer patients, with only 1 focused on prostate cancer

STUDY	PUBLICATION YEAR	COUNTRY / LANGUAGE	PARTICIPANT NUMBER	MEAN AGE	CANCER TYPE
Kim et al [42]	2016	Korea / Korean	78	44.35	Breast
McCarroll et al [43]	2015	US / English	50	58.4	Breast
Min et al [44]	2014	Korea / Korean	30	45	Breast
Sundberg et al [45]	2017	Sweden / Swedish	130	69	Prostate
Uhm et al [46]	2017	Korea / Korean	356	50.3	Breast

RESULTS CLINICAL APPROACH

4 of the 5 included studies referred to apps that assessed QoL [43- 46]

Other variables measured: depression status, daily food intake, sleep disturbance, sense of coherence, physical activity, user satisfaction, and others

All the studies allow patients to collect patient-reported outcome measures and 3 of them include a related-intervention app [43,45,46]

Adherence to the self-reporting measures was associated with higher accuracy of depression screening
Of the 3 studies that included intervention [43,45,46], only 2 reported a QoL improvement [45,46]

2 prospective nonrandomized multicenter controlled trials, 1 with control group
No RCTs

STUDY	QOL ASSESSMENT	FUNCTIONALITIES	VALIDATED QUESTIONNAIRE/TIMING	TREATMENT OFFERED	QUALITY OF STUDY
Kim et al [42]	No	PRO: daily mental health ratings over a 48-week period	PHQ-9 via app biweekly	No	Low-Medium
McCarroll et al [43]	Yes	PRO: daily, real-time, and motivational feedback + intervention	FACT-G, WEL at baseline and at 4-week follow-up	Comprehensive lifestyle program	Low-Medium
Min et al [44]	Yes	PRO: daily basis over a 90-day period	BDI, EQ-5D-3L via app on a daily basis for 90-days	No	Low-Medium
Sundberg et al [45]	Yes	PRO: daily, real-time assessment of symptoms and concerns during radiotherapy + intervention*	EORTC QLQ-C30, EORTC QLQ-PR25 via app daily at any time during radiotherapy and 3 weeks after completion	Management of symptoms	Medium-High
Uhm et al [46]	Yes	PRO + intervention*	EORTC QLQ-C30, EORTC QLQ-BR23 at baseline and 12 weeks	12-week regimen of aerobics	Medium-High

* Significant improvement in quality of life

RESULTS TECHNOLOGICAL APPROACH

2 studies involved the same app [42,44]
Only 1 app was available for download at the online store, with a free and premium version

Three of the 4 apps were targeted at cancer patients

The main features of the apps were focused on: exercise and nutrition logging; collection of PROs; detection, reporting and management of symptoms; and exercise by a step counter

App functionalities included: customization and personalization features; motivational features; and social features

STUDY	APP NAME	PLATFORM	AVAILABLE IN MARKETS	PRICE	DOWNLOADS	RATINGS	PATIENTS TARGETED
Kim et al [42]	Pit-a-Pat	Android/iOS	No	Unknown	Unknown	Unknown	Yes
McCarroll et al [43]	LoseIt!	Android/iOS	Yes	free/premium	Android: 5,000,000-10,000,000	Android: 4.4; iOS: 4.0	No
Min et al [44]	Pit-a-Pat	Android/iOS	No	Unknown	Unknown	Unknown	Yes
Sundberg et al [45]	Interaktor	Unknown	No	Unknown	Unknown	Unknown	Yes
Uhm et al [46]	Smart After Care	iOS	No	Unknown	Unknown	Unknown	Yes

DISCUSSION

- Mobile phone health apps represent an opportunity to monitor psychological distress and QoL related to cancer
- In this line, we conducted a systematic literature review
- Only **5** studies were identified with apps that focus on QoL and/or well-being assessment in breast or prostate cancer patients

Clinical and Technological Strengths and Weaknesses

The use of related-treatment mobile phone apps have reported a significantly improvement in cancer patients' QoL
Displaying daily patient reports in real time and providing personalized feedback are a significant advantage
Mobile apps are ubiquitous technologies with the potential to monitor patients and provide personalized interventions in real-time
These may take advantage of internal or external sensors to collect data

Lack of framework-based and cancer-focused apps used in studies involving cancer patients
Small samples of studies and lack of RCT protocols
Usability and accessibility issues with cancer patients

Patients' satisfaction with the health apps

Only one study provided information about satisfaction level using the app
From the market review, only one app reported a quality certification and a considerable number of user comments

More evidence-based apps are needed for breast and prostate cancer
These apps must be cancer-focused and consider usability and accessibility issues
Important to consider patient satisfaction using the app
Studies designed based on RCT are imperative for reaching high-quality evidence base for these apps

LIMITATIONS

Excluded apps that were not focused on breast or prostate cancer patients

Considered only the assessment of 2 main psychological variables in psycho-oncological care: QoL and well-being

Psychological measures, such as fatigue or the secondary symptoms produced by the cancer treatments should be considered

We might have missed some studies that were not identified with our search terms or not published

CONCLUSION

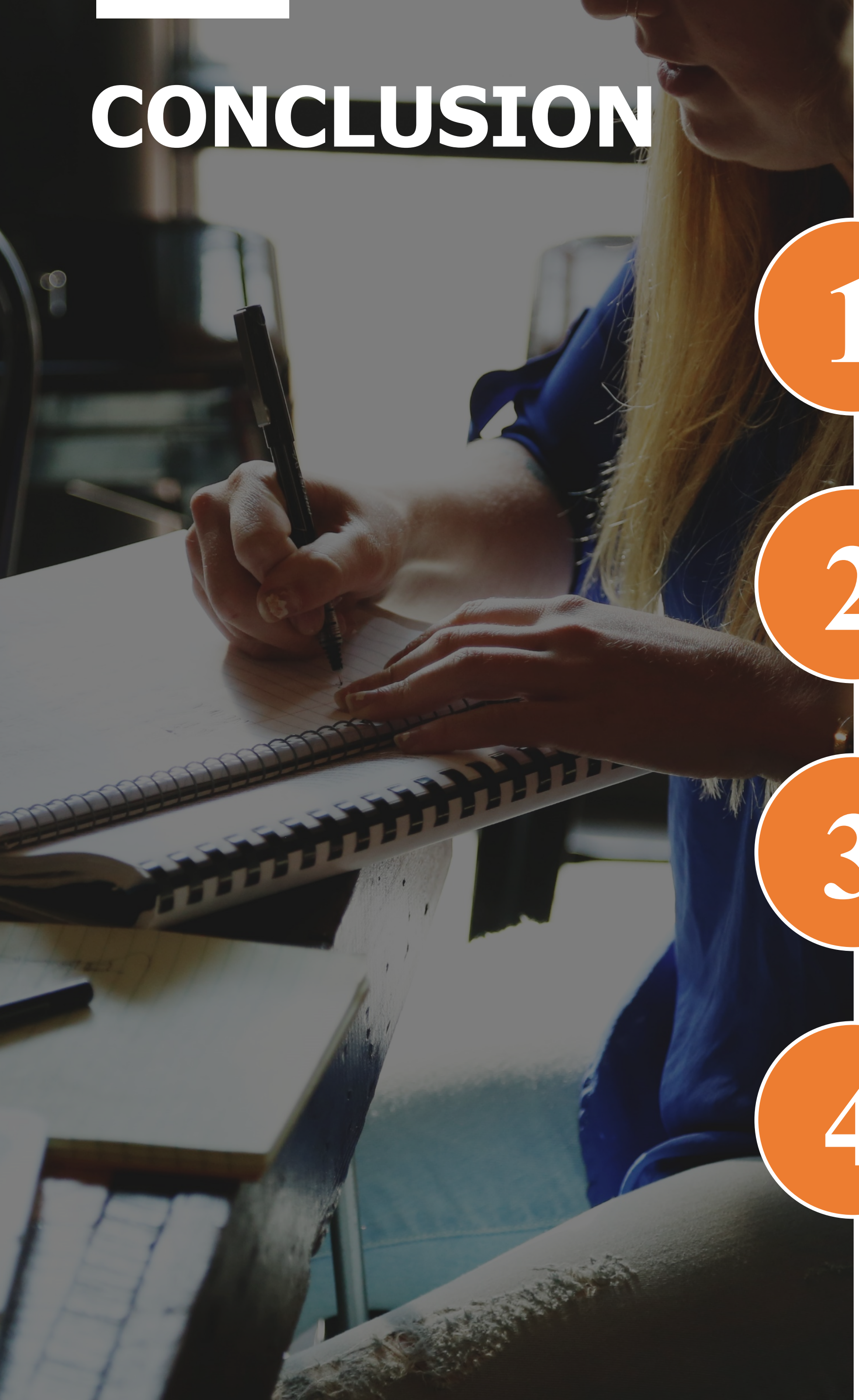

1. Lack of rigorous trials regarding QoL and/or well-being assessment in breast and/or prostate cancer patients
2. More evidence-based apps, which could be tested in futures RCT protocols, are still needed
3. Promising results are expected to be available from some RCTs that are still running
4. A strong and collective effort should be made by all health care providers to determine those cancer-focused apps that are useful and reliable for patients

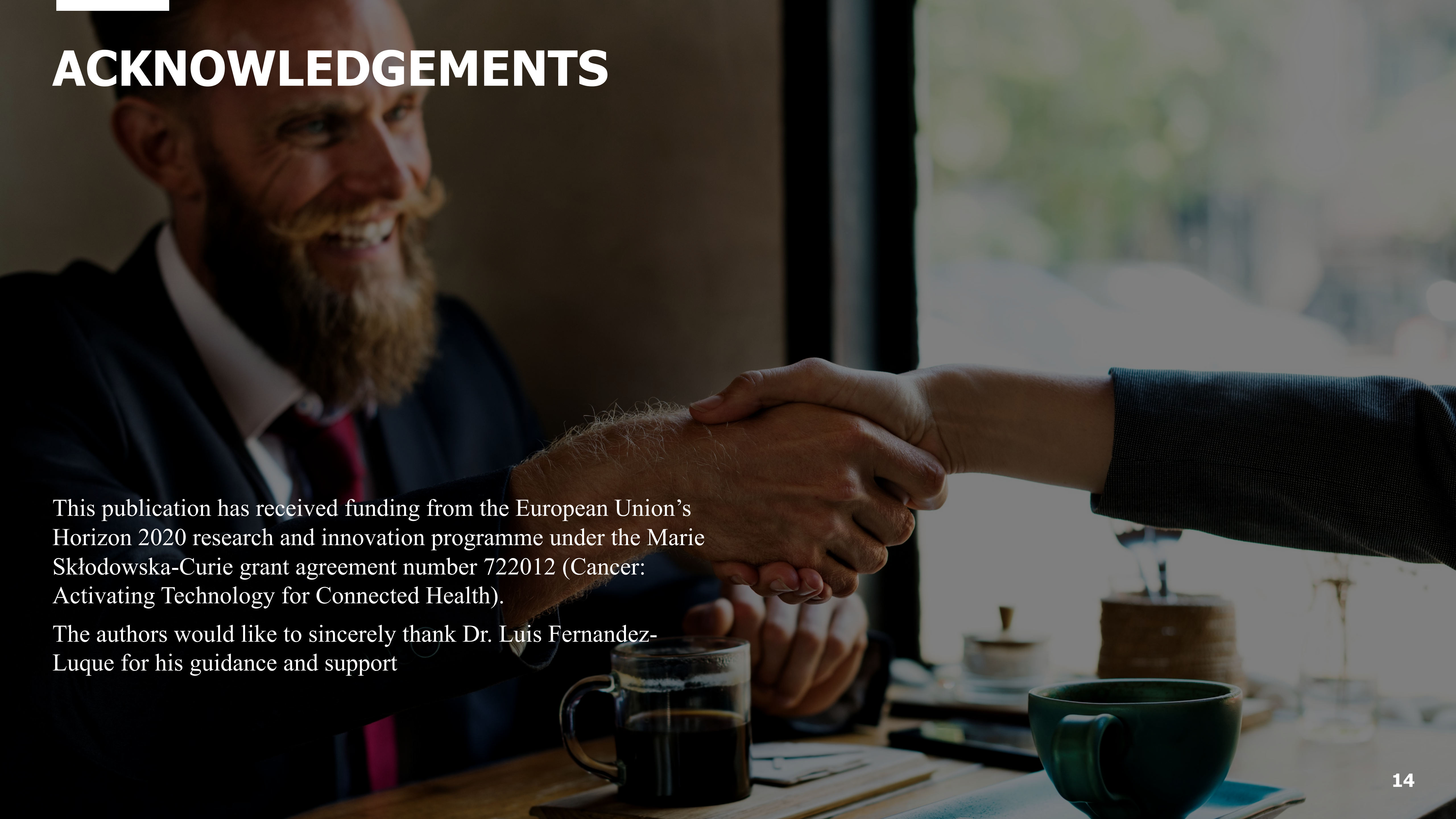

ACKNOWLEDGEMENTS

This publication has received funding from the European Union's Horizon 2020 research and innovation programme under the Marie Skłodowska-Curie grant agreement number 722012 (Cancer: Activating Technology for Connected Health).

The authors would like to sincerely thank Dr. Luis Fernandez-Luque for his guidance and support

All images used in this presentation are free stock photos licensed under Creative Commons
Source: Pexels
URL: https://www.pexels.com/
License: CC0 1.0 Universal (CC0 1.0)
License URL: https://creativecommons.org/publicdomain/zero/1.0/

This is a Multimedia Appendix to a full manuscript published in the JMIR mHealth and uHealth. For full copyright and citation information see http://dx.doi.org/10.2196/mhealth.8741
